# Biomedical Applications of Big Data and Artificial Intelligence

**DOI:** 10.3390/bioengineering12020207

**Published:** 2025-02-19

**Authors:** Yan Pei, Jijiang Yang

**Affiliations:** 1 Computer Science Division, The University of Aizu, Aizuwakamatsu 965-8580, Japan; 2 Department of Automation, Tsinghua University, Beijing 100084, China; yangjijiang@tsinghua.edu.cn

**Keywords:** artificial intelligence, big data, image processing, data mining, deep learning, bioinformatics, bioengineering, healthcare

## Abstract

This Special Issue of *Bioengineering* is dedicated to the profound impact of big data and artificial intelligence (AI) in the fields of biomedical research and healthcare. In an age defined by the rapid evolution of technology, this Issue explores the dynamic intersection of AI and data science with medicine. A total of 14 papers were accepted after a thorough review process, with their topics including disease diagnosis, medical data analysis, image processing, personalized medicine, pathological image segmentation, survival prediction, cognitive load assessment, and medical knowledge extraction. These studies aim to enhance medical image analysis, signal processing, data prediction, and interpretability to improve diagnostic accuracy, medical efficiency, and personalized treatment plans for patients. We hope the publication of this Special Issue can offer a comprehensive view of the transformative power of these innovative approaches and enrich research and investigations into the applications of big data and AI in biomedical research and healthcare.

## 1. Introduction

The convergence of big data and artificial intelligence (AI) has catalyzed transformative advancements in the biomedical field, enabling innovative solutions for complex healthcare challenges [1]. This Special Issue, dedicated to exploring the “Biomedical Applications of Big Data and Artificial Intelligence”, highlights significant advancements in research and development, such as disease diagnosis, medical data analysis, image processing, personalized medicine, pathological image segmentation, survival prediction, cognitive load assessment, and medical knowledge extraction, etc. From improving diagnostic accuracy to enhancing personalized treatment plans, these advancements underscore the potential of integrating AI and big data to revolutionize biomedical research and healthcare.

The 14 articles featured in this Special Issue, including 12 research papers and 2 review papers, encompass a wide range of topics such as healthcare big data analysis, diagnostic tools, imaging techniques, survival prediction, and explainable AI. Together, these contributions provide a comprehensive overview of how AI and big data are driving precision medicine, optimizing healthcare delivery, and advancing our understanding of complex biomedical phenomena. This editorial delves into the transformative power of these technologies and their profound implications for the future of biomedical research and healthcare.

## 2. An Overview of This Special Issue

The 14 papers accepted in this Special Issue can be categorized into four biomedical research directions: intelligent diagnosis and disease prediction, medical image processing and pathological analysis, medical signal processing and analysis, and survey and frontier research perspectives. We summarize these 14 papers from these four perspectives.

### 2.1. Intelligent Diagnosis and Disease Prediction

The critical issue of diagnostic errors in fuzzy processing, a leading cause of mortality, is addressed in the article “Trivial State Fuzzy Processing for Error Reduction in Healthcare Big Data Analysis towards Precision Diagnosis”. This study introduces a Cooperative Trivial State Fuzzy Processing method that employs fuzzy logic to mitigate uncertainties and errors in healthcare data. By grouping diagnosis-relevant and irrelevant data, the proposed method reduces trivial state errors, enhancing the precision of diagnostic outcomes. This study demonstrates how optimization-based analysis can be effectively applied to structured healthcare data, marking a significant step forward in reducing diagnostic inaccuracies.

The study “Wearable 12-Lead ECG Acquisition Using a Novel Deep Learning Approach from Frank or EASI Leads with Clinical Validation” presents an innovative approach to portable cardiovascular diagnostics. By reconstructing 12-lead ECGs using a novel deep learning model, M2Eformer, this research overcomes the limitations of traditional portable ECG devices. The validation results, including a 96% diagnostic consensus among cardiologists, underscore the clinical utility of the reconstructed ECGs. This work exemplifies the potential of AI-driven solutions in enhancing accessibility and accuracy in cardiovascular diagnostics.

The study “Personalized Explanations for Early Diagnosis of Alzheimer’s Disease Using Explainable Graph Neural Networks with Population Graphs” emphasizes the importance of explainability in AI-driven medical applications. By leveraging graph convolutional networks (GCNs) and population graphs, this research provides insights into amyloid-beta positivity prediction and the heterogeneity of Alzheimer’s disease progression. The findings highlight the potential for more nuanced diagnoses and personalized therapeutic strategies.

“GNN-surv: Discrete-Time Survival Prediction Using Graph Neural Networks” explores the application of graph neural networks (GNNs) in survival prediction for cancer patients. By leveraging patient similarity networks built from genomic and clinical data, GNN-surv achieved significant improvements in survival prediction performance compared to traditional models. This study’s adaptability across various datasets and its implications for personalized medicine underscore the transformative potential of GNNs in oncology.

The study “Prediction of Cognitive Load from Electroencephalography Signals Using Long Short-Term Memory Network” proposes an attention-based LSTM network for real-time cognitive load prediction using EEG signals. This method achieved the highest accuracy, outperforming traditional algorithms, and can enable adaptive systems to dynamically respond to users’ mental states, enhancing applications like personalized learning and fatigue detection.

### 2.2. Medical Image Processing and Pathological Analysis

In “Elucidating Multimodal Imaging Patterns in Accelerated Brain Aging: Heterogeneity through a Discriminant Analysis Approach Using the UK Biobank Dataset”, the authors explore the heterogeneity of accelerated brain aging (ABA). Utilizing multimodal imaging data and semi-supervised heterogeneity analysis (HYDRA), this study identified three distinct ABA subtypes, each associated with unique structural and functional characteristics. These findings highlight the potential for personalized neuroprotective treatments targeting age-related neurological and neuropsychiatric disorders.

The article “RGGC-UNet: Accurate Deep Learning Framework for Signet Ring Cell Semantic Segmentation in Pathological Images” addresses challenges in the diagnosis of signet ring cell carcinoma. The proposed RGGC-UNet framework incorporates residual ghost blocks and ghost coordinate attention to achieve high segmentation accuracy while minimizing computational overhead. By enriching existing datasets with annotated mask labels, this study provides a valuable resource for advancing pathological image analysis. The results demonstrate the efficacy of deep learning frameworks in enhancing diagnostic precision for complex pathological conditions.

The article “RGSB-UNet: Hybrid Deep Learning Framework for Tumor Segmentation in Digital Pathology Images” proposes a novel UNet-based architecture that combines residual ghost blocks and bottleneck transformers. This hybrid framework addresses the limitations of traditional CNN-based methods by capturing global features and achieving state-of-the-art segmentation performance. The integration of class-wise dice loss (CDL) further enhances the model’s effectiveness, as demonstrated on multiple pathology datasets. Such advancements are pivotal in automating and improving tumor segmentation processes.

“Exploring the Possibility of Measuring Vertebrae Bone Structure Metrics Using MDCT Images: An Unpaired Image-to-Image Translation Method” tackles the challenge of evaluating bone structure metrics in vivo. By employing an unpaired image-to-image translation method, this study generated micro-CT-like images from MDCT scans, enabling accurate measurement of bone metrics. The proposed approach demonstrates significant improvements in both similarity and bone structure metrics, offering a practical solution for the early diagnosis of fragility fractures.

The paper “GSN-HVNET: A Lightweight, Multi-Task Deep Learning Framework for Nuclei Segmentation and Classification” presents GSN-HVNET, a compact deep learning framework for simultaneous nuclei segmentation and classification in pathology images. By integrating novel computational blocks, it improves efficiency and accuracy compared to state-of-the-art methods. The model demonstrates practical value in pathological diagnosis by reducing redundancy and computational costs.

### 2.3. Medical Signal Processing and Analysis

The paper “Physiological Noise Filtering in Functional Near-Infrared Spectroscopy Signals Using Wavelet Transform and Long-Short Term Memory Networks” proposes a method to filter physiological noise in fNIRS signals without using the desired hemodynamic response function (dHRF). It employs wavelet transform to extract low-frequency noise components from resting-state data, which are predicted and subtracted using an LSTM network during task sessions. The technique offers a reliable alternative when traditional methods are ineffective, particularly for passive brain–computer interfaces.

The study “Named Entity Recognition of Diabetes Online Health Community Data Using Multiple Machine Learning Models” introduces the RoBERTa-BiLSTM-CRF model to identify medical entities in diabetes online health community data. Using a dataset of 1889 texts, the model achieved 81.2% accuracy and outperformed traditional models. The results demonstrate its potential for constructing medical knowledge graphs to support personalized healthcare services.

### 2.4. Survey and Frontier Research Perspectives

The two review papers in this Special Issue provide a broader perspective on the integration of big data and AI into biomedical applications, from multimodal data and AI technology [2] to machine learning and graph signal processing [3]. They discuss the current state of the art, challenges, and future directions, offering valuable insights for researchers and practitioners alike. These reviews serve as a foundation for further exploration and innovation in the field.

## 3. Conclusions

This Special Issue on the “Biomedical Applications of Big Data and Artificial Intelligence” showcases the profound impact of integrating AI and big data in healthcare. From reducing diagnostic errors and enhancing imaging techniques to enabling personalized treatments and explainable AI, the contributions highlighted here represent significant advancements in the field. These studies not only address critical challenges but also pave the way for future innovations in precision medicine and healthcare delivery.

These studies encompass areas such as intelligent diagnosis, medical image processing, signal analysis, and text mining. The key contributions of these works are as follows: First, numerous studies introduce novel models (e.g., UNet variants, GNN-surv, M2Eformer), expanding the application boundaries of AI in healthcare. Second, these studies primarily employ deep learning, graph neural networks, LSTM, and Transformer variants to enhance prediction and analysis capabilities in structured and unstructured medical data. Finally, some research concepts, such as a focus on precision medicine, personalized diagnosis, explainable AI, and automated medical image analysis, which address challenges in accuracy, efficiency, and explainability that traditional methods struggle to overcome, are implemented in these works. These studies demonstrate that AI is increasingly becoming a core driving force in biomedical computing and healthcare. Future challenges will include improving explainability, reducing computational costs, enhancing model generalization, and promoting the practical implementation of AI in clinical applications.

As we move forward into this new era of healthcare, our focus must remain on responsible innovation, patient well-being, and equitable access. The intersection of compassion and cutting-edge technology will truly unlock the potential of big data and AI in biomedical applications, making a healthier world not just a dream, but an attainable reality [4]. The diverse methodologies and applications presented in this Issue underscore the potential of interdisciplinary research in advancing biomedical science. We hope this Special Issue inspires further research and innovation in this dynamic and impactful field.
